# Identification of mitochondria-associated hub genes related to alcohol-associated liver fibrosis progression in aldehyde dehydrogenase 2 deficiency

**DOI:** 10.3389/fphys.2026.1873663

**Published:** 2026-07-13

**Authors:** Feiyu Zhang, Yanhang Gao

**Affiliations:** 1 Department of Hepatology, Center of Infectious Diseases and Pathogen Biology, The First Hospital of Jilin University, Changchun, China; 2 Jilin Provincial Key Laboratory of Metabolic Liver Diseases, The First Hospital of Jilin University, Jilin University, Changchun, China; 3 China-Singapore Belt and Road Joint Laboratory on Liver Disease Research, The First Hospital of Jilin University, Jilin University, Changchun, China

**Keywords:** ACSL1: acyl-CoA synthetase long-chain family member 1, alcohol-associated liver disease (ALD), aldehyde dehydrogenase 2 (ALDH2), mitochondria, protein-protein interaction, transcriptomics, weighted gene co-expression network analysis

## Abstract

**Background & aims:**

The pathogenesis of alcohol-associated liver fibrosis remains incompletely understood. Aldehyde dehydrogenase 2 (ALDH2) is the primary enzyme responsible for detoxifying ethanol-derived acetaldehyde. Notably, the *ALDH2* rs671 variant is carried by approximately 8% of the global population, with a prevalence of 30%–50% in East Asians. This variant reduces ALDH2 enzymatic activity. However, the contribution of impaired ALDH2 function to fibrotic susceptibility and the underlying regulatory molecules remains to be fully elucidated.

**Methods:**

Blood samples from patients with alcohol-associated liver disease (ALD) were collected for *ALDH2* rs671 genotyping, with concurrent assessment of liver stiffness. Global *Aldh2* knockout (*Aldh2^-/^*^-^) and wild-type (WT) mice were treated with ethanol or carbon tetrachloride (CCl_4_) or a combination of both.

**Results:**

Among 80 patients with ALD, those carrying the *ALDH2* rs671 variant (n = 34) exhibited comparable liver fibrosis-related indices to *ALDH2* wild-type patients (n = 46) despite significantly lower cumulative alcohol intake. Compared with WT mice, *Aldh2^-/^*^-^ mice treated with ethanol and CCl_4_ showed exacerbated hepatic fibrosis, with transcriptomic analysis revealing predominant enrichment of differentially expressed genes in mitochondrial pathways, accompanied by reduced hepatic mitochondrial size and redox imbalance. Acyl-CoA synthetase long-chain family member 1 (ACSL1) was identified as a candidate molecule associated with fibrotic progression under ALDH2-deficient conditions. ACSL1 expression was downregulated in liver tissue from ethanol plus CCl_4_-treated *Aldh2^-/^*^-^ mice, coinciding with elevated hepatic long-chain free fatty acids.

**Conclusion:**

This study suggests a potential clinical association between the *ALDH2* rs671 variant and increased fibrosis susceptibility in patients with ALD, and identifies ACSL1 as a candidate molecule associated with alcohol-associated fibrotic progression under ALDH2-deficient conditions, providing a basis for future mechanistic studies.

**Impact and implications:**

This study provides clinical evidence suggesting a potential association between the prevalent *ALDH2* rs671 variant and increased susceptibility to alcohol-associated liver fibrosis. Mitochondrial alterations may contribute to fibrotic progression in *Aldh2*-deficient mice exposed to ethanol and CCl_4_. ACSL1 was identified as a candidate molecule associated with this process. These findings extend the current understanding of mitochondrial and metabolic alterations involved in ALDH2-related alcohol-associated fibrotic injury and provide a basis for future mechanistic studies.

## Highlights

- The *ALDH2* rs671 variant may be associated with increased fibrosis susceptibility in patients with ALD.
- Mitochondrial alterations may be involved in fibrotic progression in *Aldh2*^−/−^ mice treated with ethanol and CCl_4_.
- ACSL1 is identified as a candidate molecule associated with fibrotic progression in *Aldh2*^−/−^ mice exposed to ethanol and CCl_4_.

## 1 Introduction

Alcohol-associated liver fibrosis represents a critical stage in the progression to cirrhosis and end-stage liver disease; however, current therapeutic interventions offer limited efficacy in arresting its development (Prince et al., 2023; Thursz and Lingford-Hughes, 2023). Therefore, elucidating the underlying mechanisms of the disease is essential to facilitate the development of innovative therapeutic strategies. The liver is the primary organ for alcohol metabolism, where mitochondrial aldehyde dehydrogenase 2 (ALDH2) is the key enzyme catalyzing the conversion of acetaldehyde (AcH)—a toxic ethanol (EtOH) metabolite—into non-toxic acetate. Epidemiological studies indicate that ~8% of the global population carries the *ALDH2* rs671 variant (~560 million people), whereas the prevalence reaches 30%–50% in Asians (Goedde et al., 1992; Gross et al., 2015). This variant impairs ALDH2 enzymatic activity and reduces AcH detoxification capacity, although the degree of functional impairment differs according to genotype and rs671 heterozygous carriers retain residual ALDH2 activity. Therefore, human *ALDH2* rs671 carriers should not be directly equated with complete ALDH2 deficiency. The resulting impairment of AcH clearance may promote hepatic accumulation of AcH and its toxic adducts, contributing to hepatocellular injury (Xiao et al., 2026; Gao et al., 2019). Although numerous studies have demonstrated the significance of ALDH2 in alcohol-associated liver disease (ALD), the relationship between impaired ALDH2 function, fibrosis susceptibility, and the associated regulatory molecules remains to be fully characterized.

Mitochondria serve as the primary energetic and metabolic hubs of the cell, and the disruption of their homeostasis plays a central driving role in the progression of alcohol-associated liver disease (ALD) (Gao et al., 2024; Ma et al., 2026; Goikoetxea-Usandizaga et al., 2023). Multi-omics data reveal that the hepatic transcriptome in alcohol-associated hepatitis undergoes severe reprogramming, characterized particularly by the profound dysregulation of key gene clusters, such as those encoding mitochondrial cytochrome c oxidase (Rodrigo-Torres et al., 2025; Massey et al., 2021). Concurrently, oxidative stress coupled with dysregulated xenobiotic metabolism induces aberrant post-translational modifications in mitochondrial proteins (LeFort et al., 2024). These molecular alterations compromise mitochondrial integrity, deplete cellular energy reserves, and ultimately contribute to hepatocyte injury and apoptosis. Furthermore, chronic alcohol exposure suppresses dynamin-related protein 1 expression, leading to the accumulation of maladaptive megamitochondria that exacerbate cellular injury (Ma et al., 2023; Lee et al., 2020). Mitochondrial double-stranded RNA is then delivered via exosomes to activate the toll-like receptor 3/interleukin (IL)-1β axis in hepatic macrophages (Lee et al., 2020). This mitochondria-derived damage signal subsequently stimulates a massive release of IL-17A from γδ T cells during the early stages and CD4^+^ T cells in the later stages, thereby igniting an amplified, cascading inflammatory response. The ensuing inflammatory microenvironment not only worsens hepatocyte damage but also promotes the transdifferentiation of hepatic stellate cells into myofibroblasts, accelerating extracellular matrix deposition and fibrogenesis (An et al., 2020; Torres et al., 2025). Given the pivotal role of mitochondrial dysfunction within the development of ALD, targeted mitochondrial repair demonstrates therapeutic potential (Lu et al., 2021). Current studies indicate that restoring NLR family pyrin domain containing 3-mediated mitophagy via farnesoid X receptor overexpression, or utilizing neonatal liver-derived extracellular vesicles to promote mitochondrial regeneration, can effectively halt and even reverse ALD progression (Chen et al., 2026a; Zeng et al., 2025). Collectively, the in-depth characterization and targeted rescue of mitochondrial structural and functional defects will serve as a key strategy for the future treatment of ALD.

ALDH2 is indispensable for maintaining mitochondrial homeostasis due to its exclusive localization and broad involvement in organelle metabolism. Our preliminary research has demonstrated that ALDH2 deficiency exacerbates alcohol-associated hepatocellular carcinoma by impairing mitochondria (Seo et al., 2019). Given that hepatic fibrosis is a critical precursor to carcinogenesis, we hypothesize that ALDH2 deficiency may promote fibrogenic progression by dysregulating mitochondria-related genes. Consequently, the precise identification of these genetic targets during this pathological transition is crucial for elucidating the molecular mechanisms by which ALDH2 deficiency promotes progressive liver injury.

In recent years, alongside the rapid advancement of high-throughput sequencing and bioinformatics approaches, transcriptome analysis has emerged as a powerful strategy for uncovering the molecular signatures of complex diseases (Wang et al., 2026). Notably, weighted gene co-expression network analysis (WGCNA) transcends traditional differential expression profiling by constructing discrete gene modules and correlating them with clinical phenotypes, thereby enabling the precise extraction of core regulatory networks (Ferdouse and Clugston, 2022).

Currently, the impact of impaired ALDH2 function on fibrotic vulnerability and its associated regulatory targets remains incompletely characterized. To address this gap, we integrated clinical assessments—including alcohol intake surveys and liver stiffness measurements—from patients with ALD stratified by *ALDH2* rs671 genotype. In parallel, we generated *Aldh2* global knockout (*Aldh2*^-/-^) mice and established a model of alcohol−associated liver fibrosis. Through transcriptomic sequencing and WGCNA, we systematically screened for candidate molecules. Our findings suggest a potential clinical association between the *ALDH2* rs671 variant and increased fibrosis susceptibility in patients with ALD, and demonstrate that *Aldh2* knockout exacerbates hepatic fibrosis in mice exposed to combined ethanol and carbon tetrachloride (CCl_4_) challenge. In this context, acyl-CoA synthetase long-chain family member 1 (ACSL1) was identified as a candidate molecule associated with fibrotic progression under ALDH2-deficient conditions, providing a basis for future mechanistic and translational studies.

## 2 Methods

### 2.1 Human cohort study

Patients with non-cirrhotic ALD were enrolled in this study. ALD diagnosis was based on a history of chronic alcohol consumption (> 5 years) combined with objective evidence of liver injury confirmed by biochemical assays, radiological imaging, or histological examination. The exclusion criteria comprised the following: (1) presence of viral hepatitis, autoimmune liver disease, or other co-existing liver etiologies; (2) diagnosis of severe alcoholic hepatitis [defined as a Maddrey’s Discriminant Function (MDF) score of ≥32] or hepatocellular carcinoma; (3) presence of severe hepatic inflammation or cholestasis [defined as a total bilirubin (TBIL) level > 51 μmol/L or an alanine aminotransferase (ALT) level > 5 times the upper limit of normal]; (4) incomplete data for alcohol consumption history. All participants provided written informed consent. The study protocol was approved by the Ethics Committee of The First Hospital of Jilin University (Approval No. 2024-113) and conducted in accordance with the principles of the Declaration of Helsinki.

### 2.2 Alcohol consumption assessment

Alcohol consumption data were collected using a structured, self-administered questionnaire. Alcohol intake (g/day) was estimated based on the reported drinking frequency and the amount consumed on a typical drinking day over the preceding 12 months. Drinking frequency categories were assigned median values: 0.25 for 1 time/month, 0.75 for 2–4 times/month, 2.5 for 2–3 times/week, and 4 for ≥4 times/week. The alcohol by volume was standardized at 4% for beer and 50% for spirits (e.g., Chinese Baijiu). Alcohol intake duration (years) was derived by the difference between age at baseline and age at which the participant started drinking. Cumulative alcohol intake (kg) was derived from the product of alcohol intake and alcohol intake duration.

Consistent with the World Health Organization (WHO) definitions, one standard drink was equivalent to 10 g of pure ethanol. Based on the sex-specific alcohol intake thresholds established by the National Institute on Alcohol Abuse and Alcoholism (NIAAA), individuals were classified into three drinking levels: light, moderate, and heavy drinking were defined as ≤1, 2–3, and >3 drinks/day in women and ≤2, 3–4, and >4 drinks/day in men, respectively.

### 2.3 Genotyping of the *ALDH2* rs671

Genomic DNA was extracted from peripheral blood leukocytes. The Kompetitive Allele Specific polymerase chain reaction (PCR) assay was used for genotyping of the *ALDH2* rs671 polymorphism. The reactions were conducted in a 384-well plate, with a final reaction volume of approximately 10 μL, comprising genomic DNA, 2× Master Mix, and an assay mix containing allele-specific forward primers and a common reverse primer. A passive reference dye (ROX) was included in the reaction to normalize well-to-well volume variations. Thermal cycling was performed using a standard touchdown PCR protocol: initial activation at 94 °C for 15 min, followed by a touchdown phase of 10 cycles comprising denaturation at 94 °C for 20 s and annealing/extension starting at 61 °C and decreasing by 0.6 °C per cycle for 60 s, and finally, a final amplification phase of 26 cycles at 94 °C for 20 s and 55 °C for 60 s. End-point fluorescence data for FAM and HEX fluorophores were acquired after PCR completion. Genotypes were determined by analyzing the cluster plots of normalized fluorescence intensities (FAM vs. HEX).

### 2.4 Human liver tissue samples

Human liver tissue specimens were obtained from patients with alcohol-associated cirrhosis who underwent orthotopic liver transplantation at Hospital. All patients met the clinical and histopathological diagnostic criteria for alcohol-associated liver cirrhosis. Patients with concurrent hepatic malignancies, chronic viral hepatitis (hepatitis B virus/hepatitis C virus), or other metabolic liver diseases were excluded from this study. Preoperative clinical data, including *ALDH2* genotyping, were retrieved from medical records. The study protocol was approved by the Institutional Review Board and Medical Ethics Committee. Written informed consent was obtained from all patients or their legal representatives prior to sample collection. All procedures involving human participants were conducted in strict accordance with the ethical standards of the Declaration of Helsinki. Liver tissues collected during surgery were fixed in 4% paraformaldehyde for immunohistochemical staining. The present study included four human liver tissue samples, comprising three samples from patients with the *ALDH2* wild-type genotype and one sample from an *ALDH2* rs671 heterozygous carrier.

### 2.5 Mice and mouse model

All experimental procedures used male C57BL/6 mice (age, 8–10 weeks). *Aldh2*^-/-^ mice (Stock No: NM-KO-220225) were procured from Shanghai Model Organisms Center. *Aldh2*^-/-^ mice and littermate wild-type (WT) controls were assigned to the EtOH, pair-fed + CCl_4_, or EtOH + CCl_4_ groups for 8 weeks. Mice in the EtOH-containing groups were maintained on an EtOH diet (prepared with EtOH, Cat. No. E809061, Macklin, Shanghai, China), whereas mice in the pair-fed + CCl_4_ groups received a pair-fed control diet. Mice in the CCl_4_-containing groups were intraperitoneally injected with CCl_4_ (Cat. No. C805325, Macklin) dissolved in olive oil twice weekly for 8 weeks. This study was approved by the Animal Ethics Committee of The First Hospital of Jilin University (Approval No. 2019-335) and performed in full compliance with the National Institutes of Health Guide for the Care and Use of Laboratory Animals. Animals were maintained under controlled temperature (22 °C ± 1 °C) conditions within specific pathogen-free facilities, with 12-h:12-h light–dark cycles. Water was provided ad libitum, and dietary intake was managed according to the corresponding EtOH or pair-fed regimen.

### 2.6 Bulk RNA sequencing of mouse liver tissues

OE Biotech Co., Ltd. (Shanghai, China) performed transcriptome sequencing. In brief, total RNA extraction was performed using the TRIzol reagent (Invitrogen, CA, USA), according to the manufacturer’s protocol. The NanoDrop 2000 spectrophotometer (Thermo Scientific, USA) was used for RNA quantification and purity assessments. To assess RNA integrity, the Agilent 2100 Bioanalyzer (Agilent Technologies, Santa Clara, CA, USA) was used. This was followed by library construction using VAHTS Universal V10 RNA-seq Library Prep Kit (Premixed Version), according to the manufacturer’s instructions. An Illumina Novaseq 6000 platform was used for library sequencing, followed by the generation of 150-bp paired-end reads. First, using fastp, raw reads of the fastq format were processed, and the low-quality reads were removed to obtain the clean reads, which were then mapped to the reference genome using HISAT2. Fragments per kilobase of exon per million fragments mapped were calculated for each gene, and gene-level read counts were obtained using HTSeq-count. Differential expression analysis was performed using DESeq2. A Q value of <0.05 and foldchange of >2 or <0.5 was set as the threshold to identify significantly differentially expressed genes (DEGs). To demonstrate the expression pattern of genes in different groups and samples, hierarchical cluster analysis of DEGs was performed using R (v4.2.1). Based on the hypergeometric distribution, Kyoto Encyclopedia of Genes and Genomes (KEGG) pathway and Reactome enrichment analyses of DEGs were performed using R (v4.2.1) to identify significantly enriched terms. Full RNA-seq data have been uploaded to National Center for Biotechnology Information’s Gene Expression Omnibus.

### 2.7 Transmission electron microscopy of mouse liver tissues

Liver tissues were cut into small pieces and fixed in a 2.5% glutaraldehyde-containing solution overnight at 4 °C. The samples were then processed sequentially as follows: post-fixed with 1% osmium tetroxide, stained with uranyl acetate, dehydrated using EtOH, and embedded in epoxy resin. Ultrathin sections were prepared and collected on formvar-coated copper grids, followed by contrasting with uranyl acetate and lead citrate. Finally, visual representations were captured using a Hitachi HT-7800 transmission electron microscope. For mitochondrial morphometric analysis, liver samples from three to six mice per group were evaluated. For each mouse, three to four randomly selected non-overlapping hepatocyte fields were imaged under the same magnification and acquisition settings. Mitochondrial diameter and mitochondrial area were quantified using ImageJ software. Poorly preserved regions, tissue folds, large vessels, bile ducts, and mitochondria with unclear boundaries or incomplete profiles were excluded from the analysis. The mean value from all analyzed fields was calculated for each mouse and used as one biological replicate for statistical analysis.

### 2.8 Biochemical assays in mice

Serum alanine aminotransferase (ALT) and aspartate aminotransferase (AST) levels were measured using detection kits (Cat. No. C009-3–1 and C010-3-1, respectively; Nanjing Jiancheng Bioengineering Institute, Nanjing, China). In addition, malondialdehyde (MDA), hydrogen peroxide (H_2_O_2_), and glutathione (GSH) levels in the liver homogenates and serum were measured using specific assay kits (Cat. No. A003-1-2, A064-1-1, and A006-2-1, respectively; Nanjing Jiancheng Bioengineering Institute, Nanjing, China).

### 2.9 Liquid chromatography–mass spectrometry analysis of mouse liver tissues

Frozen liver tissue samples (20–40 mg) were thawed, spiked with 20 μL of a mixed internal-standard solution containing L-2-chlorophenylalanine (4 μg/mL), succinate-D4 (2 μg/mL), L-valine-D8 (2 μg/mL), bile acid-D4 (2 μg/mL), D-Cystiferol free acid (2 μg/mL), and L-carnitine-D3 (2 μg/mL) to monitor analytical stability and technical variation, and vortexed. The samples were extracted by adding 400 μL of an ice-cold methanol: acetonitrile (2:1, v/v) solution, followed by sonication in an ice-water bath for 10 min and incubation at -20 °C for 30 min. The homogenates were then centrifuged at 13,000 rpm for 10 min at 4 °C. The transferred supernatant was vacuum-dried, reconstituted in 100 μL of a methanol: water (1:4, v/v) mixture, and centrifuged again to remove any remaining precipitates. Subsequently, the supernatant was filtered through a 0.22-μm membrane into a new vial for LC-MS.

LC-MS analysis was performed by Shanghai Luming Biological Technology Co., Ltd. Briefly, metabolite separation was achieved using a ACQUITY ultra-performance liquid chromatography I-Class Plus system equipped with an HSS T3 column (1.8 μm, 2.1 × 100 mm). The mobile phase consisted of 0.1% formic acid in water and 0.1% formic acid in acetonitrile, delivered under a standard gradient elution at 0.35 mL/min. Mass spectrometry data were acquired using a Thermo Q Exactive mass spectrometer in both positive and negative heated electrospray ionization modes. The MS parameters were set with a scan range of m/z 100–1,000, a full MS resolution of 70,000, and a data-dependent MS/MS resolution of 17,500 using stepped collision energies (10, 20, and 40 eV).

This study used a discovery-based untargeted metabolomics approach with relative quantification rather than absolute quantification. Therefore, metabolite abundance was expressed as relative LC-MS peak intensity, and no external calibration curves were generated for absolute concentration determination. Raw LC-MS data were processed using XCMS v4.5.4 for baseline filtering, peak alignment, and retention-time correction. Metabolic features with missing values (ion intensity = 0) in more than 50% of samples within a group were removed. The remaining zero values were replaced by half of the minimum detected value across the dataset, followed by log2 transformation before downstream statistical analysis.

### 2.10 Measurement of blood and hepatic acetaldehyde levels in mice

Whole blood and liver samples were collected at euthanasia, 24 h after the final CCl_4_ injection. Mice in the EtOH-treated groups were maintained on the ethanol-containing liquid diet until sacrifice. Blood and hepatic acetaldehyde levels were measured by gas chromatography–mass spectrometry (GC–MS). Briefly, 100 μL blood was mixed with 100 mg NaCl and 10 μL n-propanol. For liver samples, approximately 100 mg liver tissue was homogenized in 300 μL water containing 10 μL n-propanol using a homogenizer. The homogenates were centrifuged at 1,500 × g for 5 min at 4 °C, followed by a brief low-speed centrifugation for 30 s. The resulting supernatant was carefully transferred into a new headspace vial, which was immediately sealed with a crimper before GC–MS analysis. To minimize acetaldehyde loss, samples were processed rapidly using pre-chilled materials and kept at 4 °C or on ice during preparation.

### 2.11 Histological analysis of mouse liver tissues and immunohistochemistry staining of human and mouse liver tissues

For hematoxylin and eosin (H&E) staining, paraffin-embedded sections were dewaxed in xylene, stained with hematoxylin for 35 s, and stained with eosin for 140 s. Standard protocols were used for Masson’s trichrome staining. For IHC, sections underwent heat-induced antigen retrieval in citrate buffer (Cat. No. 005000, Invitrogen, CA, USA). Endogenous peroxidase activity was quenched by incubating with 3% H_2_O_2_, sections were blocked with 3% normal goat serum buffer for 1 h at room temperature. The sections were then incubated with primary antibodies overnight at 4 °C, which was followed by incubation with secondary antibodies (Cat. No. 8814S or 8125S, Cell Signaling Technology, Danvers, MA USA) at room temperature for 1 h. The ImmPACT 3,3′-diaminobenzidine (DAB) Substrate Kit (Cat. No. ZK1018, Vector 2 Laboratories, CA, USA) was used for visualizing the stained sections. The following primary antibodies were used: α-smooth muscle actin (Cat. No. ab124964, Abcam, Cambridge, UK) and Acyl CoA synthetase 1 (Cat. No. PB10025, Boster, Wuhan, China). The Olympus BX43 microscope was used for image acquisition. For quantitative analysis of Masson’s trichrome and IHC staining, five randomly selected non-overlapping fields at 100× magnification and five randomly selected non-overlapping fields at 200× magnification were captured from each mouse liver section under identical microscope and camera settings. Large vessels, bile ducts, tissue folds, and damaged areas were avoided during field selection. Quantification was performed using ImageJ software (National Institutes of Health, Bethesda, MD, USA) with the same threshold settings applied to all images within each staining assay. Masson’s trichrome staining was quantified as the percentage of collagen-positive area relative to the total tissue area, whereas IHC staining was quantified as the percentage of DAB-positive area relative to the total tissue area. The average value of the analyzed fields was used as the staining level for each mouse, and four to five mice per group were included for statistical analysis.

### 2.12 Total RNA isolation and reverse transcription quantitative PCR of mouse liver tissues

Total RNA extraction from liver tissues or cell lysates was performed using TRIzol reagent (Cat. No. R401-01-AA, Vazyme, Nanjing, China). To reverse transcribe RNA into single-stranded cDNA, the High-Capacity cDNA Reverse Transcription Kit (Cat. No. 2816898, Thermo Fisher Scientific, Waltham, MA, USA) was used. Gene expression was quantified *via* qPCR using ChamQ Universal SYBR qPCR Master Mix (Cat. No. 7E751K3, Vazyme, Nanjing, China) on a QuantStudio 5 Real-Time PCR System (Thermo Fisher Scientific, Waltham, MA, USA). The mRNA levels of 18s RNA were used as an internal control. The relative mRNA expression was calculated using the 2^−ΔΔCt^ method. The primer sequences used for RT-qPCR are provided in **Supplementary Table 1**. For the RT-qPCR validation panel, *P* values were adjusted for multiple testing using the Benjamini–Hochberg false discovery rate (FDR) method.

### 2.13 Western blotting of mouse liver tissues

Liver tissues and cells were homogenized or lysed in radioimmunoprecipitation assay buffer (Cat. No. YH374135, Thermo Fisher Scientific, Waltham, MA, USA) containing Halt Protease and Phosphatase Inhibitors (Cat. No. 78447, Thermo Fisher Scientific, Waltham, MA, USA). The protein samples were loaded into polyacrylamide gels (Cat. No. 220A019, Absin, Shanghai, China) and transferred to nitrocellulose membranes (Cat. No. 0000208128, Merck, Darmstadt, Germany). After blocking with 1% bovine serum albumin, the nitrocellulose membranes were incubated overnight with primary antibodies at 4 °C. After washing with Tris-buffered saline containing 0.1% Tween 20 (TBST), the membranes were incubated with anti-rabbit or anti-mouse immunoglobulin G horseradish peroxidase-linked secondary antibody (Cat. No. 7074S or 7076S, Cell Signaling Technology, Danvers, MA, USA), and protein bands were visualized using SuperSignal Maximum Sensitivity Substrate (Cat. No. WG328673, Thermo Fisher Scientific, Waltham, MA, USA). The following antibodies were used: β-actin (Cat. No. 66009-1-Ig, Proteintech, Wuhan, China). ALDH2 (Cat. No. ab133306, Abcam, Cambridge, UK).

### 2.14 Construction of WGCNA and module identification using mouse liver transcriptomic data

To identify co-expression modules associated with the severe fibrotic phenotype observed in EtOH + CCl_4_-treated *Aldh2*^−/−^ mice, WGCNA was performed on the RNA-seq datasets using the “WGCNA” R package. Because WGCNA and DESeq2 were based on the same expression dataset and partly captured overlapping group-level transcriptional signals, WGCNA was used as an exploratory tool for candidate gene prioritization. Fragments Per Kilobase Million (FPKM) values were first transformed using log2(FPKM + 1). Genes with low expression variability across all samples, defined as a standard deviation ≤ 0.2 in the log2-transformed expression matrix, were removed. The remaining genes from 15 samples were used for subsequent WGCNA analysis. First, sample clustering was conducted to identify potential outliers and assess sample heterogeneity. The “goodSamplesGenes” function was then used to check the expression matrix and remove genes or samples with excessive missing values, if present. The “pickSoftThreshold” function was used to evaluate the scale-free topology fit index and mean connectivity across a range of soft-thresholding powers. Because the scale-free topology fit index did not reach the recommended threshold, sample clustering was further examined and showed no obvious single-sample outlier. The separation of EtOH + CCl_4_-treated *Aldh2*^−/−^ samples was consistent with their severe fibrotic phenotype; therefore, a soft-thresholding power of 18 was empirically selected to construct a signed co-expression network according to the WGCNA recommendations for datasets with fewer than 20 samples (Langfelder and Horvath, 2017). The adjacency matrix, based on Pearson correlation coefficients, was subsequently transformed into a topological overlap matrix to evaluate network interconnectedness. Modules were identified via hierarchical clustering with a minimum module size of 25 and a merging threshold (cut height) of 0.25. For module–trait correlation analysis, the severe fibrotic phenotype was encoded as a binary trait, with EtOH + CCl_4_-treated *Aldh2*^−/−^ mice assigned a value of 1 and the other three groups assigned a value of 0. Modules showing the strongest positive and negative correlations with this binary trait were selected as key modules, and their genes were retained for further candidate gene screening.

### 2.15 Identification of mitochondria-associated genes in mouse liver transcriptomic data

Mouse mitochondrial genes were obtained from the mouse MitoCarta 3.0 database, which contains 1,140 genes encoding mouse mitochondrial proteins.

### 2.16 Construction of the protein–protein interaction network

A PPI network associated with mitochondria-associated genes was constructed using the STRING database (v12.0), with the organism restricted to *Mus musculus* and the confidence score threshold set at ≥ 0.4 to ensure reliability. The network was visualized and analyzed using Cytoscape (v3.10.3). To identify key biological components, the Molecular Complex Detection (MCODE) plugin was first used to detect densely connected functional modules (subnetworks) based on vertex density. Subsequently, the cytoHubba plugin was employed to evaluate node centrality using four representative topological algorithms with different network emphases: Maximal Clique Centrality (MCC), Density of Maximum Neighborhood Component (DMNC), Betweenness, and Edge Percolated Component (EPC).

To assess the influence of cutoff selection, cutoff-sensitivity analysis was performed by extracting the Top 10, Top 15, and Top 20 ranked genes from each of the four cytoHubba algorithms. At each cutoff threshold, genes shared by the four cytoHubba-ranked gene sets were identified and then compared with genes in MCODE cluster 1. Genes consistently retained across different cutoff thresholds and located within MCODE cluster 1 were prioritized as hub genes.

### 2.17 Statistical analysis

Statistical analysis and data presentation were performed using GraphPad Prism 9 (GraphPad Software, San Diego, CA, USA) and R software version 4.2.1 (R Foundation for Statistical Computing, Vienna, Austria). Data are presented as the mean ± standard deviation (SD). The normality of the data distribution was assessed using the Shapiro–Wilk test. Adjusted clinical analyses were performed using multivariable linear regression to account for potential confounders. For between-group comparisons, statistical significance was determined using a two-sided unpaired t-test for normally distributed data or using the Mann–Whitney U test for non-normally distributed data. For comparisons among three or more groups, one-way analysis of variance followed by Tukey’s *post-hoc* test was employed for normally distributed data. For non-normally distributed data, the Kruskal–Wallis test followed by Dunn’s multiple comparison test was used. The sample sizes (n) are provided in figure legends. Statistical significance was defined as *P* < 0.05. Exact *P* values are provided in the figures (*P < 0.05, **P < 0.01, and ***P < 0.001).

## 3 Results

### 3.1 Clinical association between the *ALDH2* rs671 variant and fibrosis-related indices in patients with ALD

**Figure 1:**
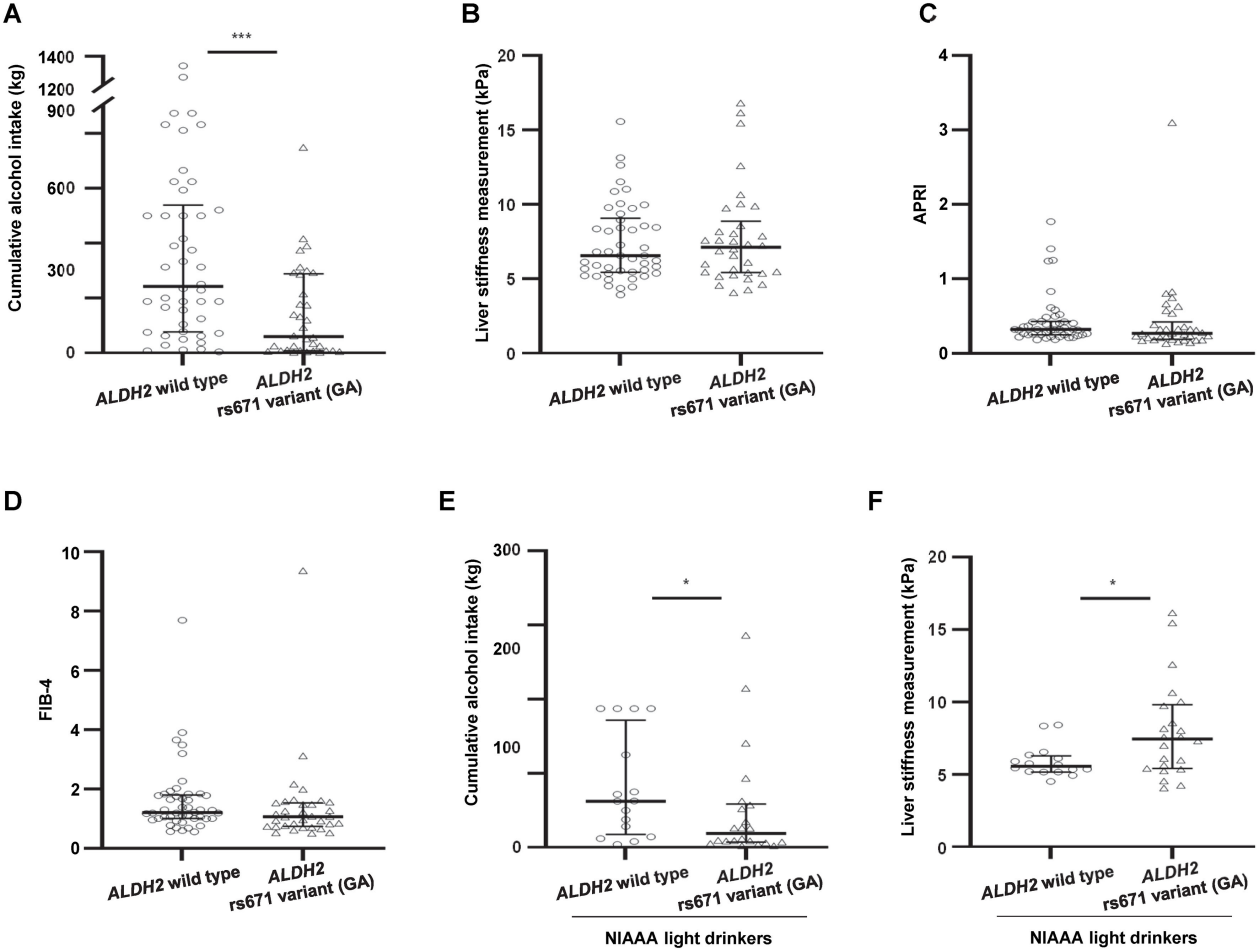
Stratification analysis of cumulative alcohol intake and liver fibrosis-related indices based on the *ALDH2* rs671 genotype in patients with non-cirrhotic ALD. **(A)** Cumulative alcohol intake in patients with non-cirrhotic ALD according to the *ALDH2* rs671 genotype. The overall cohort includes wild-type subjects (n = 46) and *ALDH2* rs671 variant (GA) carriers (n = 34). **(B–D)** liver stiffness measurement **(B)**, APRI **(C)**, and FIB-4 **(D)** in the total non-cirrhotic ALD population across different genotypes. **(E)** Cumulative alcohol intake among a subgroup of non-cirrhotic ALD patients classified as light drinkers according to the NIAAA criteria. This subset includes wild-type subjects (n = 16) and *ALDH2* rs671 variant (GA) carriers (n = 22). **(F)** liver stiffness measurement within the NIAAA-defined light drinking subgroup. Panels **(E, F)** represent exploratory and hypothesis-generating subgroup analyses. *P < 0.05, **P < 0.01, and ***P < 0.001. ALD, alcohol-associated liver disease; ALDH2, aldehyde dehydrogenase 2; APRI, AST-to-platelet ratio index; FIB-4, fibrosis-4 index; GA, heterozygous ALDH2 rs671 genotype; NIAAA, National Institute on Alcohol Abuse and Alcoholism.

To explore the potential clinical relevance of ALDH2 impairment in alcohol-associated liver fibrosis, we analyzed 80 non-cirrhotic patients with ALD stratified by *ALDH2* rs671 genotype. Their baseline characteristics are presented in **Table 1**. All patients included in this cohort were male, with a median age of 47 years (IQR, 42–56 years). The cohort comprised 46 patients in the *ALDH2* wild-type group and 34 patients carrying the *ALDH2* rs671 variant, all of whom were heterozygous. Age and liver function indices, including ALT, AST, gamma-glutamyl transferase (GGT), and TBIL, were comparable between the two groups, whereas body mass index (BMI) and controlled attenuation parameter (CAP) were significantly higher in patients carrying the *ALDH2* rs671 variant. As shown in **Figure 1A**, the median cumulative alcohol intake was significantly lower in patients carrying the *ALDH2* rs671 variant than in wild-type subjects (54.6 kg [IQR, 9.2–176.2 kg] *vs*. 241.8 kg [IQR, 84.5–514.8 kg], *P* < 0.001). Despite this markedly lower alcohol exposure, the median liver stiffness measurement (LSM) values were comparable between the two groups (7.1 kPa [IQR, 5.4–8.9 kPa] *vs*. 6.5 kPa [IQR, 5.5–8.8 kPa], *P* = 0.911; **Figure 1B**). To address the potential confounding effects of BMI and CAP, multivariable linear regression was further performed using ln-transformed LSM as the dependent variable. After adjustment for BMI and CAP, *ALDH2* rs671 genotype was not significantly associated with ln-transformed LSM (β = 0.069, 95% confidence interval: −0.091 to 0.229, *P* = 0.393). Furthermore, as shown in **Figures 1C, D**, no statistically significant differences were observed in APRI or FIB-4 values between the two groups. These findings suggest that patients carrying the *ALDH2* rs671 variant showed comparable fibrosis-related indices despite substantially lower cumulative alcohol exposure, supporting a cautious interpretation of a potential association between the *ALDH2* rs671 variant and increased susceptibility to alcohol-associated liver fibrosis.

**Table 1:** Baseline characteristics of patients with alcohol-associated liver disease stratified by *ALDH2* rs671 genotype.

		*ALDH2* rs671 genotypes			
Variables	Overall (N = 80)	Wild-type (GG) (n=46, 57.5%)	Variant (GA) (n = 34, 42.5%)	W/χ2	*P*
Age, Median (IQR)	47 (42-56)	52 (47-58)	45 (42-55)	640.5	0.170
Male, n (%)	80 (100.0)	46 (100.0)	34 (100.0)		
BMI (kg/m²), Median (IQR)	25.1 (23.2-27.7)	24.2 (22.8-27.3)	26.4 (24.0-29.7)	1025.5	**0.018**
CAP (dB/m), Median (IQR)	273.5 (250.4-294.9)	265.5 (246.6-284.3)	290.5 (265.4-303.0)	1063.0	**0.006**
LSM (kPa), Median (IQR)	7.1 (5.4-9.7)	6.5 (5.5-8.8)	7.1 (5.4-8.9)	794.0	0.911
Alcohol consumption patterns					
Type of beverage, n (%)				5.048	0.080
Beer	11 (13.8%)	3 (6.5%)	8 (23.5%)		
Spirits	40 (50.0%)	26 (56.5%)	14 (41.2%)		
Mixed	29 (36.3%)	17 (37.0%)	12 (35.3%)		
Drinking frequency, n (%)				450.0	**< 0.001**
1 time/month	14 (17.5%)	3 (6.5)	11 (32.4)		
2–4 times/month	11 (13.8%)	8 (17.4)	3 (8.8)		
2–3 times/week	20 (25.0%)	7 (15.2)	13 (38.2)		
≥ 4 times/week	35 (43.8%)	28 (60.9)	7 (20.6)		
Alcohol intake (g/day), median (IQR)	66.5 (36.0-85.0)	80.0 (42.0-100.0)	55.0 (31.5-73.5)	554.5	**0.027**
Standard Drinks Per Week (drinks/week), median (IQR)	13.8 (5.0-24.0)	20.0 (12.0-32.0)	12.5 (4.2-22.1)	1106.5	**0.004**
Alcohol Intake Duration (years), median (IQR)	20 (10-30)	30 (20-30)	20 (10-30)	487.5	**0.003**
Cumulative alcohol intake (kg), median (IQR)	104.0 (37.4-299.5)	241.8 (84.5-514.8)	54.6 (9.2-176.2)	405.5	**< 0.001**
NIAAA				557.0	**0.018**
Light Drinking	38 (47.5%)	16 (34.8%)	22 (64.7%)		
Moderate Drinking	19 (23.8%)	14 (30.4%)	5 (14.7%)		
Heavy Drinking	23 (28.8%)	16 (34.8%)	7 (20.6%)		
Laboratory parameters, median (IQR)					
ALT (U/L)	29.5 (19.8-43.1)	28.8 (20.2-40.2)	30.5 (14.8-47.8)	763.5	0.861
AST (U/L)	28.4 (21.1-36.7)	29.4 (21.7-37.2)	27.6 (20.4-37.9)	699.0	0.422
GGT (U/L)	64.5 (36.5-109.1)	77.6 (41.2-145.0)	64.3 (32.6-107.3)	614.5	0.104
ALP (U/L)	78.4 (64.4-95.7)	79.8 (68.0-95.5)	74.4 (64.9-88.8)	758.5	0.823
Albumin (g/L)	45.1 (43.9-47.2)	45.2 (43.3-47.2)	45.1 (44.0-46.6)	771.5	0.923
Total bilirubin (µmol/L)	14.3 (11.4-19.0)	15.4 (11.3-20.2)	13.7 (11.6-18.5)	775.5	0.953
Total bile acid (µmol/L)	2.4 (1.5-3.6)	2.8 (1.7-3.6)	2.5 (1.7-3.8)	785.0	0.981
Platelet count (10^9^/L)	225.0 (198.0-256.0)	223.0 (191.0-237.8)	227.5 (196.5-293.5)	959.5	0.085
TG (mmol/L)	2.1 (1.2-3.3)	2.5 (1.2-3.9)	1.8 (1.3-2.8)	400.5	0.133
LDL-C (mmol/L)	3.2 (2.7-3.8)	3.4 (2.8-4.0)	3.3 (3.0-3.8)	516.5	1.000
Creatinine (µmol/L)	78.5 (72.1-85.0)	76.3 (71.5-84.8)	81.7 (76.8-90.7)	325.0	0.342
APRI	0.357 (0.260-0.486)	0.366 (0.284-0.487)	0.307 (0.216-0.440)	610.0	0.095
FIB-4	1.176 (0.855-1.641)	1.203 (0.997-1.791)	1.048 (0.741-1.528)	580.5	0.069

Data are presented as median (IQR) or n (%), as appropriate. The *ALDH2* rs671 genotypes were classified as the wild-type genotype (GG) and the heterozygous genotype (GA). W/χ² indicates the Wilcoxon rank-sum test statistic or chi-square test statistic, as appropriate. ALDH2, Aldehyde Dehydrogenase 2; ALP, Alkaline Phosphatase; ALT, Alanine Aminotransferase; APRI, Aspartate Aminotransferase to Platelet Ratio Index; AST, Aspartate Aminotransferase; BMI, Body Mass Index; CAP, Controlled Attenuation Parameter; FIB-4, Fibrosis-4 Index; GGT, Gamma-glutamyl Transferase; IQR, Interquartile Range; LDL-C, Low-density Lipoprotein Cholesterol; LSM, Liver Stiffness Measurement; NIAAA, National Institute on Alcohol Abuse and Alcoholism; TG, Triglyceride.

Bold *P* values indicate statistical significance (*P* < 0.05).

Considering that carriers of the *ALDH2* rs671 variant typically consume less alcohol because of reduced ALDH2 enzymatic activity, the cohort was stratified into light, moderate, and heavy drinking groups based on the NIAAA criteria. Most carriers of the *ALDH2* rs671 variant were classified into the light drinking group. Subgroup analysis of these NIAAA-defined light drinkers is summarized in **Supplementary Table 2**. Given that the primary comparison of LSM in the full cohort did not reach statistical significance, this subgroup analysis was considered exploratory and hypothesis-generating. Within this subgroup, baseline clinical parameters, including age, liver function indices, BMI, and CAP, were comparable between the two genotype groups. As shown in **Figure 1E**, the median cumulative alcohol intake remained significantly lower in variant carriers than in *ALDH2* wild-type patients (18.7 kg [IQR, 7.6–55.9 kg] *vs*. 62.2 kg [IQR, 24.6–140.4 kg], *P* = 0.049). In contrast, LSM values were significantly higher in variant carriers than in *ALDH2* wild-type patients (7.4 kPa [IQR, 5.5–9.4 kPa] *vs*. 5.6 kPa [IQR, 5.2–6.2 kPa], *P* = 0.039; **Figure 1F**). These data suggest a possible association between the *ALDH2* rs671 variant and increased susceptibility to alcohol-associated liver fibrosis, particularly among patients meeting the criteria for NIAAA-defined light drinking.

### 3.2 ALDH2 deficiency exacerbates hepatic fibrosis in mice under combined ethanol and CCl_4_ challenge

**Figure 2:**
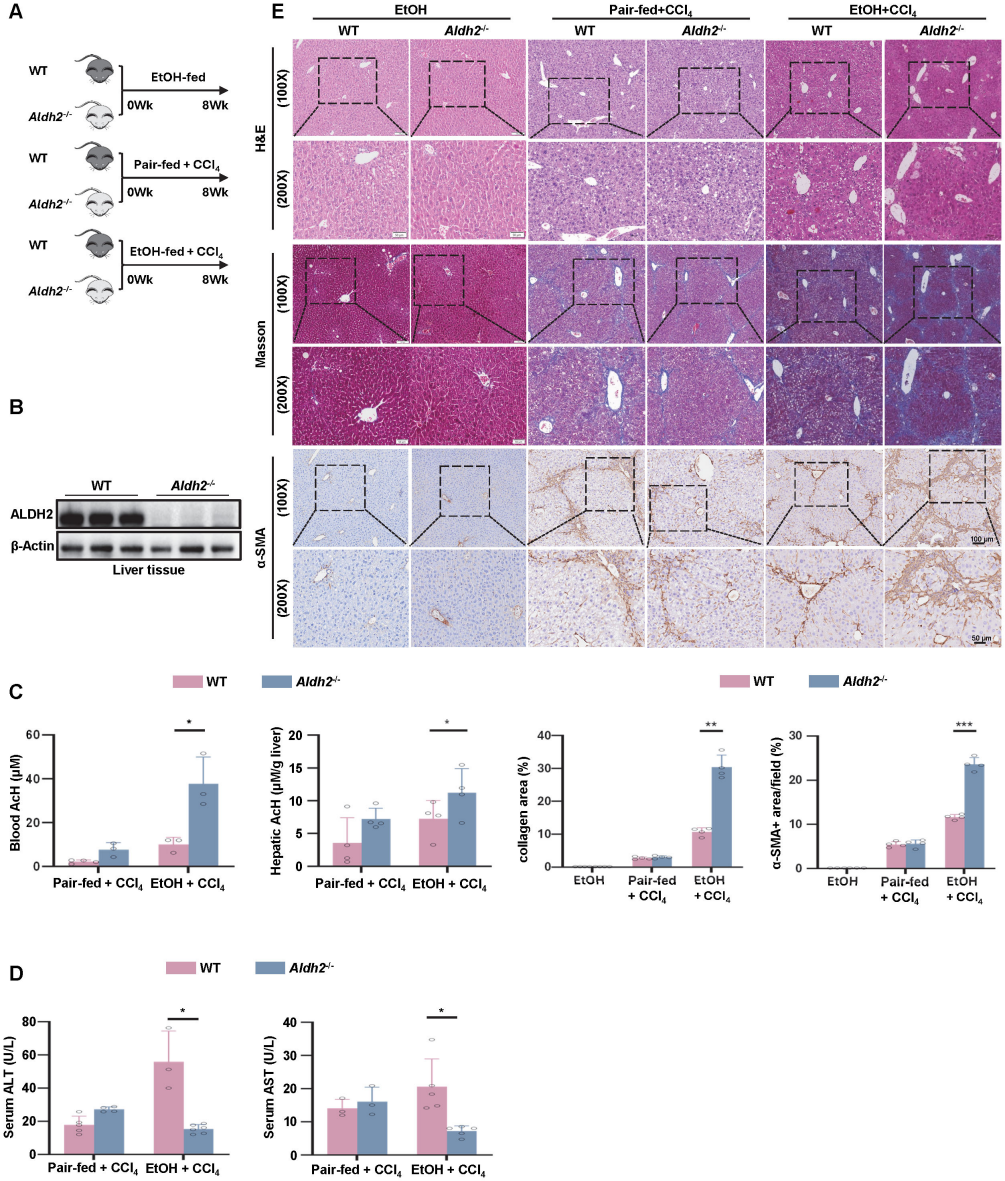
EtOH-fed *Aldh2^-/^*^-^ mice exhibited exacerbated CCl_4_-induced liver fibrosis. **(A)** Schematic of the *in vivo* study design. WT and *Aldh2*^-/-^ mice were divided into three groups: (1) EtOH diet; (2) pair-fed diet + CCl_4_ (i.p.); and (3) EtOH diet + CCl_4_ (i.p.). Mice (n = 6–8 per group) were treated for 8 weeks and euthanized 24 h after the final injection. **(B)** Hepatic ALDH2 levels were measured by Western blotting in WT and *Aldh2^-/^*^-^ mice (n = 3 mice per group). **(C)** Blood and hepatic AcH levels (n = 3–4 mice per group). **(D)** Serum ALT and AST levels (n = 3–6 mice per group). **(E)** Representative images of liver sections stained with H&E, Masson’s trichrome, and α-SMA immunohistochemistry. Collagen deposition and α-SMA immunostaining were quantified (n = 4 mice per group). *P < 0.05, **P < 0.01, and ***P < 0.001. AcH, acetaldehyde; ALDH2, aldehyde dehydrogenase 2; *Aldh2^-/^*^-^ mice: Aldehyde dehydrogenase 2 knockout mice, ALT, alanine aminotransferase; AST, aspartate aminotransferase; CCl_4_, carbon tetrachloride; EtOH, ethanol; H&E, hematoxylin and eosin; i.p., intraperitoneal injection; α-SMA, α-smooth muscle actin; WT, wild-type.

To investigate the role of ALDH2 deficiency in alcohol-associated hepatic fibrogenesis, a whole−body *Aldh2*^-/-^ mouse model was generated, and the experimental scheme is shown in **Figure 2A**. Successful knockout was confirmed by the absence of ALDH2 expression in the liver tissues of *Aldh2*^-/-^ mice (**Figure 2B**). Moreover, under EtOH + CCl_4_ treatment, AcH concentrations in both peripheral blood and liver tissue were markedly higher in *Aldh2*^-/-^ mice than in WT controls (**Figure 2C**). Serum ALT and AST levels were quantified (**Figure 2D**). Histopathological analysis revealed that ethanol exposure alone did not induce detectable hepatic fibrosis in either WT or *Aldh2*^-/-^ mice. In the pair-fed plus CCl_4_ groups, WT and *Aldh2*^-/-^ mice showed comparable collagen deposition and α-SMA-positive areas. In contrast, after combined EtOH and CCl_4_ treatment, *Aldh2*^-/-^ mice exhibited significantly increased collagen deposition and expanded α-SMA-positive regions compared with WT mice (**Figure 2E**). These findings suggest that ALDH2 deficiency exacerbates hepatic fibrosis in mice under combined EtOH and CCl_4_ challenge.

### 3.3 DEGs are enriched in mitochondrial pathways in *Aldh2^-/^*^-^ mice treated with ethanol and CCl_4_

**Figure 3:**
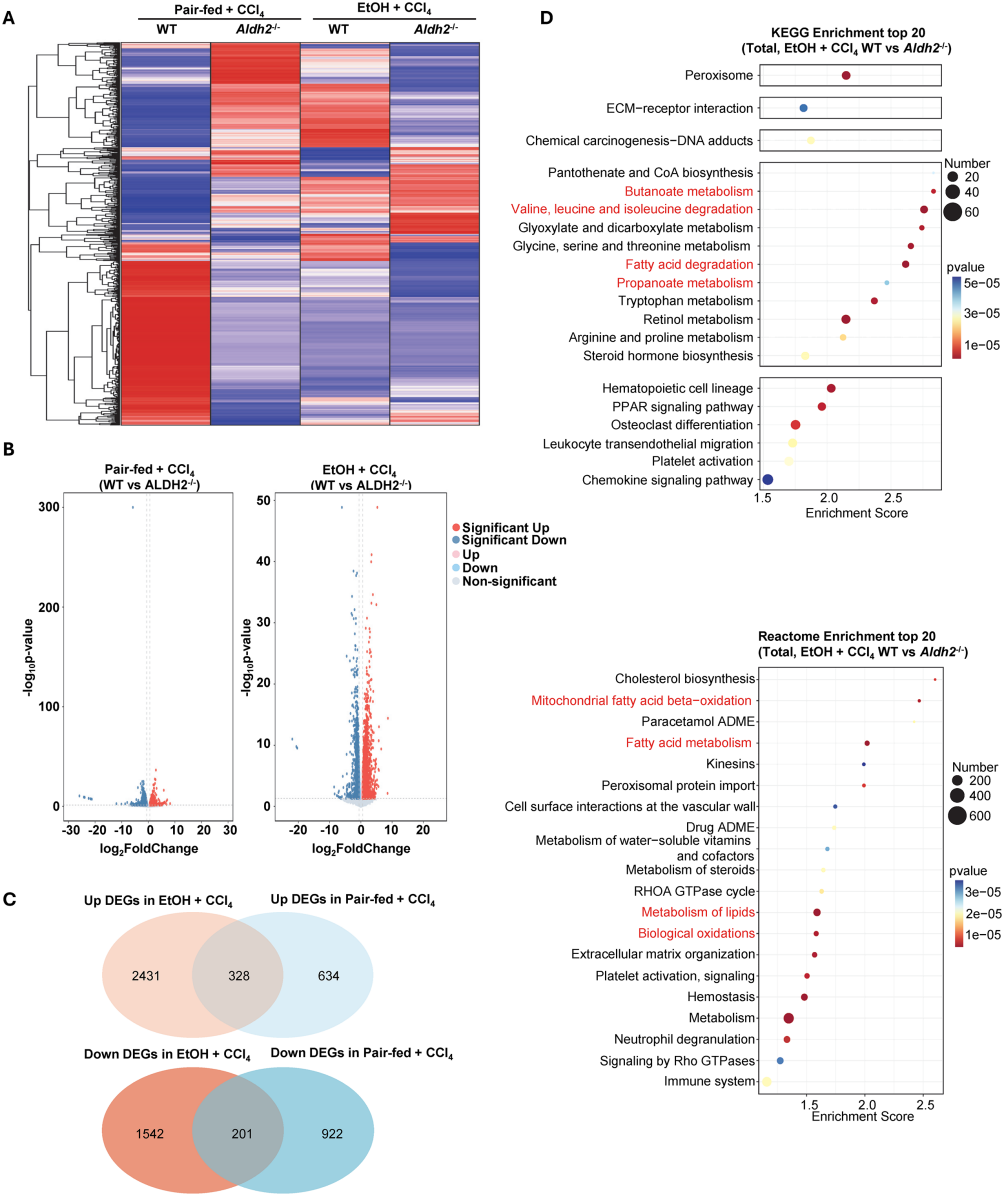
Identification and functional enrichment analysis of DEGs in mouse liver tissues. Bulk RNA-sequencing was performed on 15 mouse liver samples: pair-fed + CCl_4_ WT (n = 3), pair-fed + CCl_4_ *Aldh2*^−/−^ (n = 3), EtOH + CCl_4_ WT (n = 5), and EtOH + CCl_4_ *Aldh2*^−/−^ (n = 4). **(A)** Heatmap of DEGs across the experimental groups. **(B)** Volcano plots of DEGs between WT and *Aldh2*^-/-^ mice in the pair-fed + CCl_4_ and EtOH + CCl_4_ groups. **(C)** Venn diagram showing the overlap among DEG sets. **(D)** Top 20 enriched KEGG and Reactome pathways. CCl_4_, carbon tetrachloride; DEGs, differentially expressed genes; EtOH, ethanol; KEGG, Kyoto Encyclopedia of Genes and Genomes; WT, wild-type.

To further characterize hepatic transcriptomic alterations associated with ALDH2 deficiency in CCl_4_-based fibrogenic models with or without ethanol feeding, liver samples from WT and *Aldh2*^-/-^ mice in the pair-fed plus CCl_4_ and EtOH plus CCl_4_ groups were subjected to bulk RNA-seq analysis. The analysis identified a significant increase in the number of differentially expressed transcripts in the *Aldh2*^−/−^ mice, with 2759 transcripts showing significant upregulation and 1743 transcripts showing significant downregulation compared to the WT mice after 8 weeks of EtOH + CCl_4_ treatment. And in pair-fed + CCl_4_ group, 962 genes were significantly upregulated and 1123 were downregulated in *Aldh2*^−/−^ mice relative to WT controls. More interestingly, alcohol feeding dramatically altered the profile of gene expression in *Aldh2*-deficient mice (**Figures 3A–C**). To investigate the functional implications of the DEGs between WT and *Aldh2*^−/−^ mice in the EtOH + CCl_4_ group, we performed pathway enrichment analysis using the KEGG and Reactome databases. As shown in **Figure 3D**, the analysis revealed that multiple top-ranked pathways were predominantly associated with mitochondrial function and metabolic processes. These transcriptomic findings suggest that mitochondria-associated alterations may be involved in the aggravated hepatic fibrosis observed in *Aldh2*^−/−^ mice following EtOH + CCl_4_ treatment.

### 3.4 ALDH2 deficiency is associated with mitochondrial structural abnormalities and redox imbalance in EtOH-exposed mouse livers

**Figure 4:**
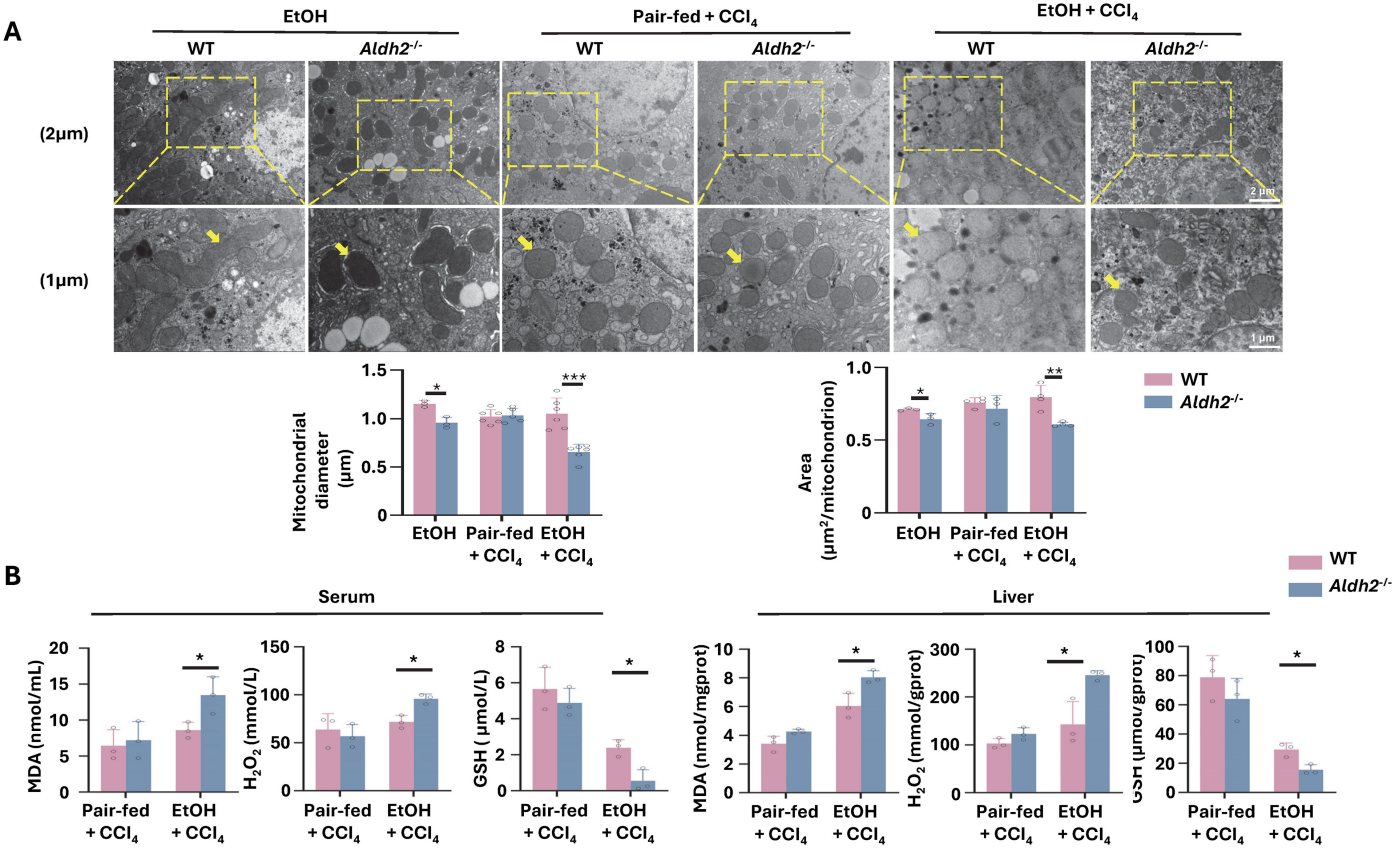
ALDH2 deficiency promotes hepatic mitochondrial fragmentation and oxidative stress in mice under EtOH exposure. **(A)** Representative TEM images showing hepatocyte mitochondria and quantification of average mitochondrial diameter and area (n = 3–6 mice per group). Yellow arrows indicate mitochondria. **(B)** Levels of MDA, H_2_O_2_, and GSH in serum and mouse liver tissues (n = 3 mice per group). *P < 0.05, **P < 0.01, and ***P < 0.001. ALDH2, aldehyde dehydrogenase 2; TEM, transmission electron microscopy; AcH, acetaldehyde; MDA, malondialdehyde; H_2_O_2_, hydrogen peroxide; GSH, glutathione.

Given that the DEGs were enriched in multiple mitochondrial pathways, we next examined hepatocyte mitochondrial morphology by TEM and assessed oxidative stress-related markers to further clarify the impact of ALDH2 deficiency on hepatic mitochondria. In the pair-fed + CCl_4_ group, mitochondrial diameter and area did not differ significantly between WT and *Aldh2^-/^*^-^ mice. However, in the EtOH and EtOH + CCl_4_ groups, both parameters were significantly reduced in *Aldh2^-/^*^-^ mice than in WT mice (**Figure 4A**), suggesting increased mitochondrial fragmentation under EtOH exposure conditions. We then assessed hepatic MDA, GSH, and H_2_O_2_ levels. In the pair-fed + CCl_4_ groups, ALDH2 deficiency did not significantly affect MDA, GSH, or H_2_O_2_ levels. In contrast, in the EtOH + CCl_4_ group, hepatic MDA, GSH, and H_2_O_2_ levels were significantly increased in *Aldh2^-/^*^-^ mice compared with WT mice, indicating that ALDH2 deficiency exacerbates oxidative stress and redox imbalance in this setting (**Figure 4B**). Taken together, the enrichment of DEGs in multiple mitochondrial pathways, combined with the observed mitochondrial ultrastructural abnormalities and enhanced oxidative stress, suggests a potential involvement of mitochondrial alterations in ALDH2 deficiency–exacerbated alcohol-associated liver fibrosis under combined EtOH and CCl_4_ challenge.

### 3.5 Identification of mitochondria-associated hub genes associated with fibrosis progression

**Figure 5:**
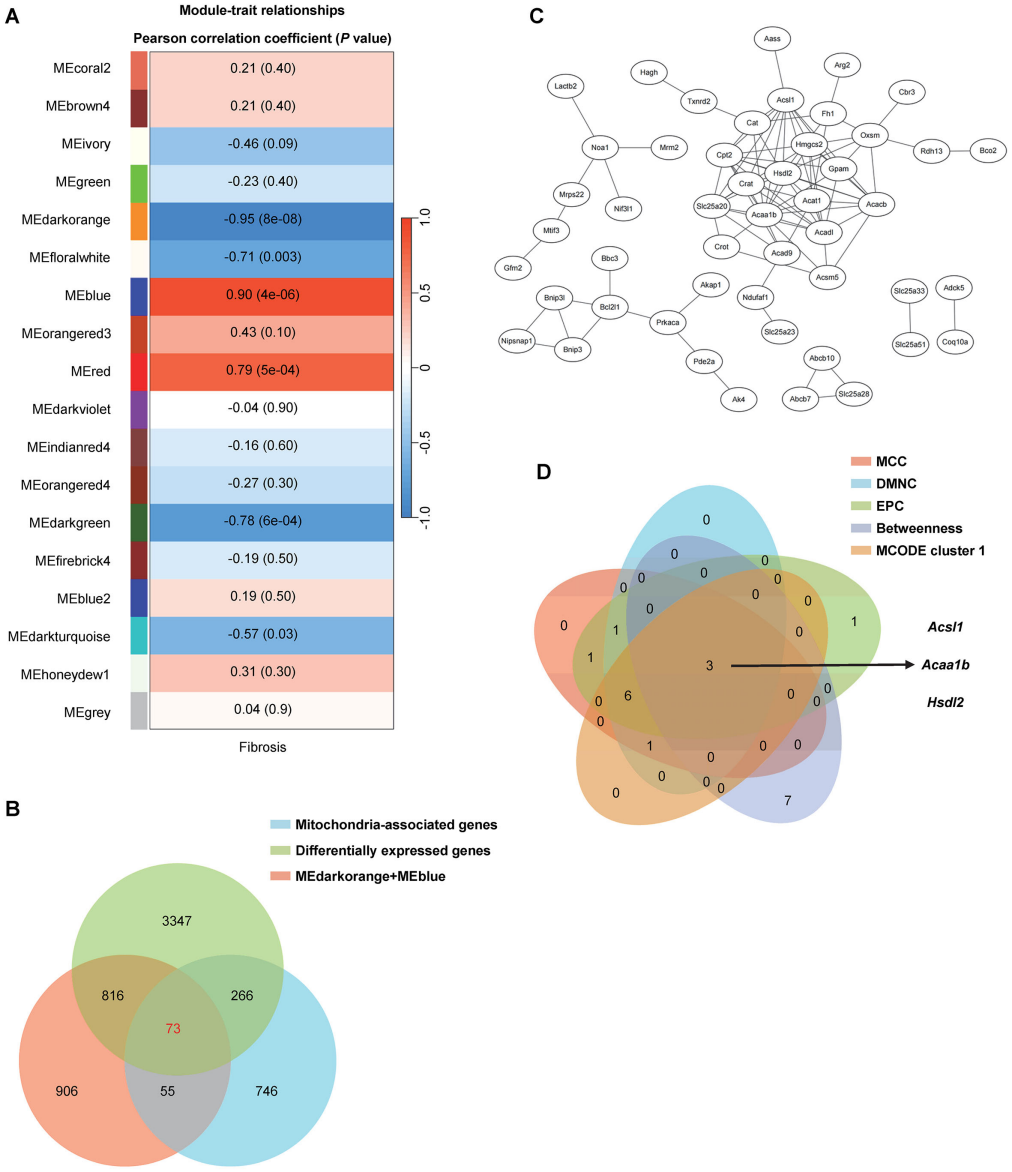
Identification of hub genes through integrated analysis of mouse liver transcriptomic data. **(A)** Correlation heatmap depicting the associations between identified gene modules and the fibrosis-related trait based on mouse liver RNA-seq samples (n = 15). Each cell displays the Pearson’s correlation coefficient and the corresponding *P*-value. **(B)** Venn diagram showing the overlap among genes in the dark orange and blue modules, DEGs, and mitochondria-related genes. **(C)** PPI network construction of the candidate genes. **(D)** Venn diagram showing the overlap among the Top 10 ranked genes from four cytoHubba algorithms (MCC, DMNC, EPC, and Betweenness) and genes in MCODE cluster 1. DEGs, differentially expressed genes; DMNC, density of maximum neighborhood component; EPC, edge percolated component; MCC, maximal clique centrality; MCODE, molecular complex detection; PPI, protein–protein interaction.

Given the mitochondrial alterations observed in ALDH2 deficiency–exacerbated fibrotic progression, we sought to identify mitochondria-associated hub genes related to fibrosis progression. WGCNA identified 18 distinct co-expression modules (**Supplementary Figure 1A**). Among them, the MEblue module exhibited the strongest positive correlation with the fibrosis-related trait (correlation = 0.90, *P* < 0.001), while the MEdarkorange module showed the most significant negative correlation with this trait (correlation = −0.95, *P* < 0.001) (**Figure 5A** and **Supplementary Figure 1B**). These two modules, containing 1501 and 349 genes, respectively, were selected for subsequent analysis. By intersecting the genes from these two modules with DEGs and mitochondria-related genes, we refined the selection to a core set of candidate genes (**Figure 5B**). A PPI network was then constructed to further prioritize these candidates (**Figure 5C**). The network comprised 49 nodes and 73 edges, with an average node degree of 2.63 and an average local clustering coefficient of 0.441. Subsequent MCODE and cytoHubba analyses identified three overlapping hub genes, *Acsl1*, *Acaa1b*, and *Hsdl2* (**Figure 5D**).

### 3.6 Identification of ACSL1 as a candidate molecule associated with fibrotic progression in ALDH2 deficiency

**Figure 6:**
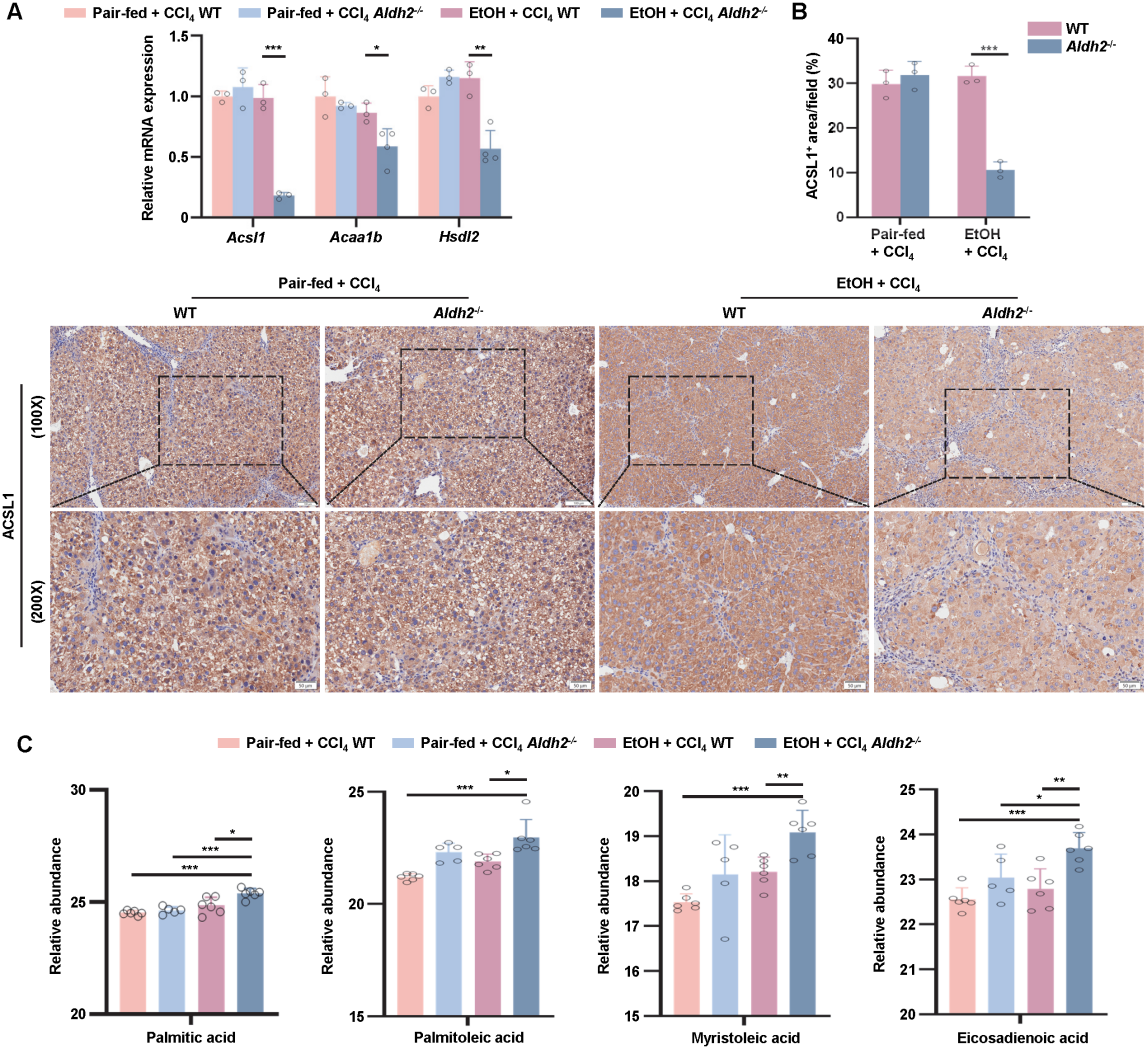
Experimental validation of ACSL1 expression in mice. **(A)** RT-qPCR analysis of the hepatic mRNA expression levels of the selected hub genes in mice (n = 3–4 mice per group). **(B)** Representative immunohistochemical images and corresponding quantification of ACSL1 staining in mouse liver sections (n = 3 mice per group). **(C)** LC-MS analysis of hepatic FFAs in mice (n = 5–6 mice per group). *P < 0.05, **P < 0.01, and ***P < 0.001. ACSL1, acyl-CoA synthetase long-chain family member 1; ALDH2, aldehyde dehydrogenase 2; FFAs, free fatty acids; IHC, immunohistochemistry; LC-MS, liquid chromatography–mass spectrometry; RT-qPCR, reverse transcription-quantitative polymerase chain reaction.

To validate the expression of the identified hub genes, RT-qPCR was performed on hepatic tissues harvested from the mouse models. The results demonstrated that the transcriptional levels of these hub genes were generally consistent with the transcriptome sequencing data, among which *Acsl1* exhibited the most pronounced alteration (**Figure 6A**). Therefore, ACSL1 was selected for further expression-level validation. Consistently, IHC staining of liver sections revealed that the ACSL1-positive area was significantly reduced in *Aldh2*^−/−^ mice treated with ethanol and CCl_4_ compared with the other groups (**Figure 6B**). To further explore the potential translational relevance of this finding, ACSL1 expression was examined in human liver specimens as an exploratory analysis. IHC staining showed that the ACSL1-positive area was lower in liver tissue from one patient with alcohol-associated cirrhosis carrying the heterozygous *ALDH2* rs671 genotype than in liver tissues from *ALDH2* wild-type patients (n = 3) (**Supplementary Figure 2**). Given previous reports that ACSL1 deficiency promotes the accumulation of free fatty acids (FFAs), we further quantified hepatic FFA levels via LC-MS. The relative abundance of palmitic acid, palmitoleic acid, myristoleic acid, and eicosadienoic acid were increased in ethanol- and CCl_4_-treated *Aldh2*^−/−^ mice compared with the other groups, showing an inverse trend relative to the ACSL1-positive area (**Figure 6C**). In summary, these findings suggest that ACSL1 is a candidate molecule associated with aggravated alcohol-associated liver fibrosis under ALDH2-deficient conditions.

## 4 Discussion

Our study presents clinical evidence suggesting a potential association between the *ALDH2* rs671 variant and increased susceptibility to alcohol-associated liver fibrosis. Using an *Aldh2*^−/−^ mouse model, we observed mitochondrial alterations that may be associated with ALDH2 deficiency–exacerbated liver fibrosis under combined EtOH and CCl_4_ challenge. Through integrated analyses and validation, ACSL1 was identified as a candidate molecule associated with fibrotic progression under ALDH2-deficient conditions.

Our clinical findings provide exploratory human context suggesting a possible association between the *ALDH2* rs671 variant and increased susceptibility to alcohol-associated liver fibrosis, which is consistent with our hypothesis derived from previous research. The *ALDH2* rs671 polymorphism is the most common genetic variant in East Asian populations and represents one of the most prevalent enzymatic deficiencies worldwide. Previous work demonstrated that, under combined alcohol and CCl_4_ challenge, ALDH2 deficiency promotes the progression of alcohol-associated hepatocellular carcinoma (HCC) (Seo et al., 2019). Notably, liver fibrosis is a critical stage in the progression of chronic liver diseases toward end-stage malignancies, and this process is strongly influenced by pathological alterations in the hepatic microenvironment (Gao et al., 2024).

In the present study, ALDH2 deficiency exacerbated hepatic fibrosis in mice exposed to combined ethanol and CCl_4_ challenge. This model was selected because ethanol exposure alone generally induces steatosis and inflammation in mice, and although prolonged or intensified ethanol-feeding regimens may produce some degree of hepatic fibrosis, they often do not generate robust and reproducible fibrotic changes (Bertola et al., 2013; Cao et al., 2026; Xu et al., 2015). Previous studies have also shown that combined ethanol and CCl_4_ treatment induces substantial hepatic fibrosis and inflammatory changes and partially recapitulates the relationship among fibrosis, inflammation, and hepatocyte proliferation observed in human ALD (Brol et al., 2019). Thus, our findings support a role for ALDH2 deficiency in aggravating fibrotic progression in an ethanol-associated, CCl_4_-accelerated fibrogenic setting. Notably, EtOH + CCl_4_-treated *Aldh2*^-/-^ mice showed lower serum ALT/AST levels than WT mice, despite more severe fibrosis. This finding suggests that serum aminotransferase levels may be dissociated from chronic fibrosis progression. Previous studies have reported that alcohol-associated chronic liver injury models can develop hepatic inflammation and fibrosis despite relatively lower serum ALT/AST levels (Xu et al., 2015). Similar findings have also been observed in non-alcoholic fibrosis models, such as thioacetamide-induced fibrosis in *Prmt6*-deficient mice (Schonfeld et al., 2023). Moreover, ALDH2 deficiency itself may alter aminotransferase responses to alcohol exposure, as low ALT levels have been reported in alcohol-exposed ALDH2-deficient humans and mice (Seike et al., 2025; Matsumoto et al., 2014). Therefore, in our study, serum aminotransferases may not fully capture the cumulative process of extracellular matrix deposition and fibrotic tissue remodeling.

In this study, pathway enrichment analysis of DEGs in the mouse liver suggested that mitochondria-related pathways may be involved in ALD, corroborating previous reports (Rodrigo-Torres et al., 2025; Seo et al., 2019; Xu et al., 2025; Thoudam et al., 2023). For instance, longitudinal paired liver biopsies and transcriptome profiling in patients with alcoholic hepatitis revealed profound transcriptomic reprogramming during disease progression, characterized by a marked dysregulation of mitochondrial cytochrome c oxidase-related gene clusters. This mitochondrial transcriptional disruption directly drives hepatocyte senescence, which in turn triggers the senescence-associated secretory phenotype and accelerates pathological advancement (Rodrigo-Torres et al., 2025). Furthermore, Gene Ontology enrichment analysis in *Aldh2*-deficient mouse models of alcohol-associated fatty liver has highlighted energy metabolism pathways, specifically those governing mitochondrial function and intercellular signaling (Xu et al., 2025). Collectively, these findings suggest that mitochondrial alterations may contribute to the aggravation of ALD under ALDH2-deficient conditions.

In addition to mitochondria-related pathways, the enrichment analysis shown in **Figure 3D** also identified immune and inflammatory pathways, such as chemokine signaling and leukocyte transendothelial migration. Previous studies have demonstrated that innate and adaptive immune responses are involved in the pathogenesis and progression of alcohol-associated liver disease, in which inflammatory mediators, chemokines, macrophages, and neutrophils contribute to sustained hepatic inflammation (Shen et al., 2025). Chemokines such as C-C motif chemokine ligand 2 and C-X-C motif chemokine ligand 1/2 in experimental models, as well as IL-8 in human alcohol-associated liver disease, have been implicated in immune-cell recruitment and inflammatory amplification during alcohol-associated liver injury (Shen et al., 2025). In addition, interactions between inflammatory cells and hepatic non-parenchymal cells, particularly macrophage–hepatic stellate cell crosstalk, may promote extracellular matrix remodeling and fibrogenesis (Patidar et al., 2024). Therefore, these findings suggest that immune-cell recruitment, inflammatory activation, and vascular–immune interactions may also contribute to the progression of alcohol-associated liver fibrosis.

Our further bioinformatic analysis and validation demonstrated that in alcohol-fed *Aldh2*-deficient mice, ACSL1 was significantly downregulated at both the transcriptional and translational levels, accompanied by a substantial accumulation of FFAs. This suggests that ACSL1 may be associated with this pathological process. These findings are consistent with previous reports demonstrating that alcohol exposure significantly decreases the transcriptional level of *Acsl1* in mouse livers (Clugston et al., 2011). Furthermore, it has been established that the loss of ACSL1 leads to mitochondrial dysfunction and excessive accumulation of FFAs (Deng et al., 2025). As a central component in maintaining lipid homeostasis, the acyl-CoA synthetase long-chain (ACSL) family catalyzes the conversion of FFAs into fatty acyl-CoA to initiate downstream lipid metabolism (Grevengoed et al., 2014). Among the four ACSL isoenzymes expressed in the liver (ACSL1, 3, 4, and 5), ACSL1 is the predominant subtype, accounting for approximately 50% of the total hepatic ACSL activity (Klett et al., 2017; Lin et al., 2025). ACSL1 can effectively utilize saturated fatty acids containing 10–16 carbon atoms and unsaturated fatty acids with 20–26 carbon atoms for fatty acid oxidation, triglyceride synthesis, and phospholipid production (Wright et al., 2024; Han et al., 2023). In alcohol-associated fatty liver, alcohol suppresses ACSL1 expression by inhibiting the signal transducer and activator of transcription 5 signaling pathway, which subsequently drives the lysosomal translocation of BCL2-associated X/p-mixed lineage kinase domain-like pseudokinase, resulting in increased lysosomal membrane permeabilization and the activation of lysosomal cell death programs (Dong et al., 2023). Interestingly, however, the regulatory effect of alcohol on ACSL1 expression appears to be organ-specific. In contrast to its inhibitory effect in the liver, chronic ethanol exposure has been shown to specifically upregulate ACSL1 expression in microglia, ultimately leading to cognitive impairment (Hao et al., 2026).

While the role of ACSL1 in alcohol-associated liver fibrosis remains largely unexplored, it has been reported to exert protective effects in fibrosis across multiple other organs. In pulmonary fibrosis, ACSL1 mitigates disease progression by attenuating mitochondrial damage and activating PTEN-induced kinase 1/Parkin-dependent mitophagy (Lin et al., 2024). In the kidney, the miR-130a-3p/ACSL1 axis restricts lipid deposition, thereby alleviating tubulointerstitial fibrosis (Jiang et al., 2024). Furthermore, in dimethylnitrosamine-induced liver fibrosis, aberrant upregulation of miR-34c in hepatic stellate cells suppresses ACSL1 expression. This leads to the depletion of intracellular lipid droplets, which triggers the activation of quiescent hepatic stellate cells and the subsequent secretion of collagen, ultimately aggravating fibrogenesis (Li et al., 2021).

Beyond *Acsl1*, other hub genes identified in this study (*Acaa1b and Hsdl2)* may also be linked to mitochondrial homeostasis and fibrotic progression. Although our present experimental validation focused primarily on ACSL1, these remaining candidates may warrant future mechanistic exploration. Previous studies have shown that, in a fibrosis-related experimental setting, ACSL1 can attenuate mitochondrial damage, reduce reactive oxygen species accumulation, and preserve mitochondrial membrane potential (Lin et al., 2024). HSDL2 depletion has also been reported to reduce mitochondrial branching and increase mitochondrial fragmentation, suggesting a possible role in mitochondrial structural regulation (Chen et al., 2026b). In addition, *Acaa1b* has been implicated in mitochondrial metabolism-related transcriptional changes in lung injury and post-injury lung fibrosis, suggesting a potential association with mitochondrial metabolic remodeling in fibrotic contexts (Samson et al., 2024). Therefore, the links between these hub genes and mitochondrial homeostatic imbalance, as well as their potential contribution to alcohol-associated liver fibrosis under ALDH2-deficient conditions, remain to be further clarified.

This study has several limitations. First, the clinical findings were observational and based on non-invasive fibrosis assessments. Given the non-significant primary LSM comparison in the full cohort and the possibility of residual confounding, the NIAAA-defined light-drinking subgroup analysis should be interpreted as exploratory and hypothesis-generating. Second, the *Aldh2*^−/−^ mouse model represents complete ALDH2 deficiency and cannot fully recapitulate the heterozygous *ALDH2* rs671 status observed in human carriers. Third, although EtOH-only groups were included for histopathological evaluation, they were not included in the RNA-seq analysis; therefore, the transcriptomic differences between *Aldh2*^−/−^ and WT mice cannot be fully separated from CCl_4_-related injury severity or potential cytochrome P450 family 2 subfamily E member 1 (CYP2E1)-mediated amplification of CCl_4_ toxicity. Fourth, in the WGCNA analysis, the relatively low scale-free topology fit index introduces uncertainty into the identified co-expression modules. Finally, the validation of ACSL1 expression in human liver tissues was limited by the small sample size; therefore, these findings should be interpreted as preliminary and supportive rather than conclusive. In addition, the mechanistic role of ACSL1 in fibrogenesis remains to be clarified. Future studies using larger clinical cohorts, more human liver specimens, *Aldh2* E487K knock-in models, and functional experiments are warranted.

In conclusion, our findings suggest a possible clinical association between the *ALDH2* rs671 variant and increased fibrosis susceptibility in patients with ALD, and identify ACSL1 as a candidate molecule associated with alcohol-associated fibrotic progression under ALDH2-deficient conditions.

## Data Availability

The datasets presented in this study can be found in online repositories. The names of the repository/repositories and accession number(s) can be found below: https://www.ncbi.nlm.nih.gov/, GSE317551.
