# Supplementary material for: Identification of mitochondria-associated hub genes related to alcohol-associated liver fibrosis progression in aldehyde dehydrogenase 2 deficiency
Source: Front Physiol. 2026 Jul 13;17:1873663. doi: 10.3389/fphys.2026.1873663 (PMC13402135; doi:10.3389/fphys.2026.1873663)
Supplement: Supplementary file 1 [file DataSheet1.docx]

***Supplementary Material***

**Supplementary Figure**

**
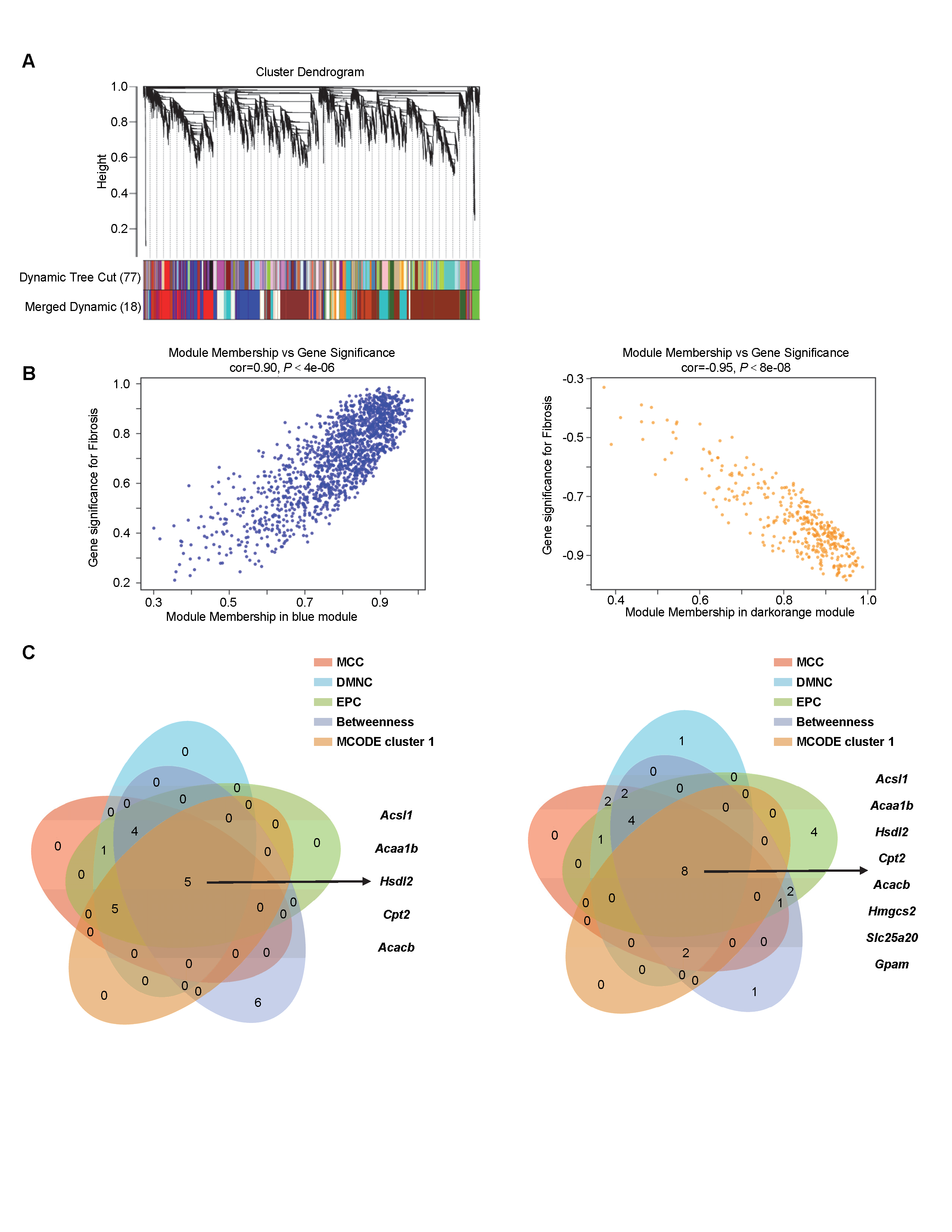
**

**Supplementary Figure S1. Identification of hub genes.** (A) Cluster dendrogram. (B) Scatter plot of genes in the blue and darkorange modules, with gene significance on the y-axis and module membership on the x-axis. (C) Venn diagram showing the overlap among the Top 15 (left) and TOP 20 (right) ranked genes from four cytoHubba algorithms (MCC, DMNC, EPC, and Betweenness) and genes in MCODE cluster 1. Abbreviations: MCODE: Molecular complex detection, MCC: Maximal clique centrality, DMNC: Density of Maximum Neighborhood Component, EPC: Edge Percolated Component.

**
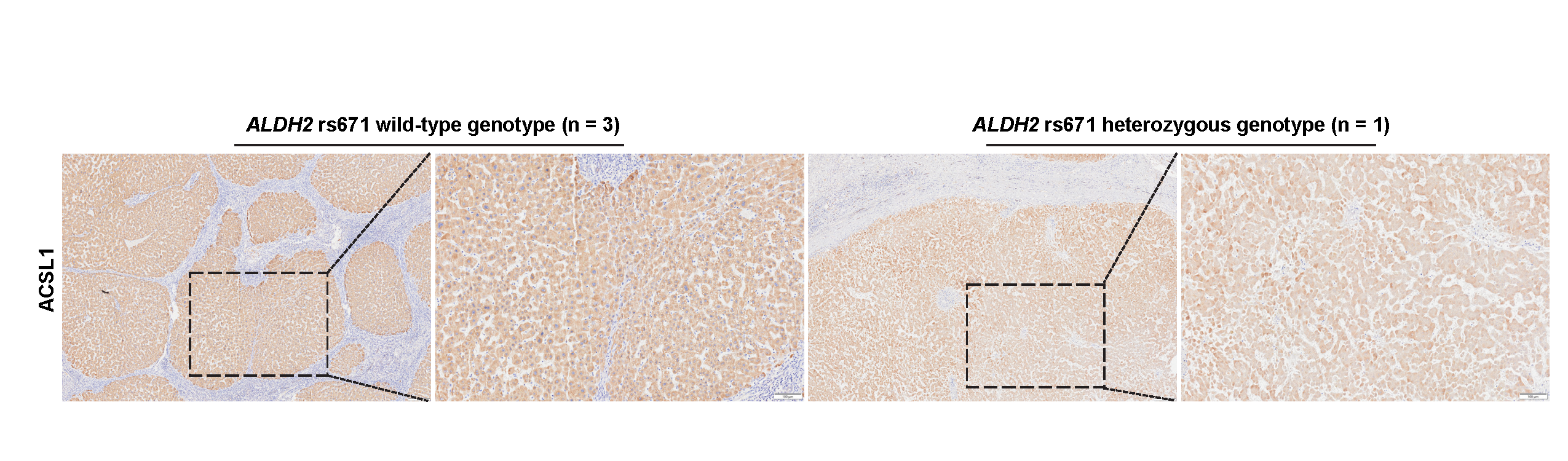
**

**Supplementary Figure S2.** **Exploratory observation of hepatic ACSL1 expression in patients with alcohol-associated cirrhosis.** Representative IHC images of ACSL1 in liver sections from patients with alcohol-associated cirrhosis carrying the *ALDH2* rs671 wild-type genotype (n = 3) and from a patient carrying the *ALDH2* rs671 heterozygous genotype (n = 1). Abbreviations: ACSL1, acyl-CoA synthetase long-chain family member 1; ALDH2, aldehyde dehydrogenase 2; IHC, immunohistochemistry.

Supplementary Table

**Supplementary Table S1**. Sequences of primers used for qPCR assay in mice

| Genes  (mouse) | Forward primer | Reverse primer |
| --- | --- | --- |
| *Acsl1* | ATCAGGCTGCTTATGGACGACC | CCAACAGCCATCGCTTCAAGGA |
| *Acaa1b* | GGAGAATGTGGCTGAGCGGTTT | AGGACAGTGGTTGTCACAGGCA |
| *Hsdl2* | CCGAAACTCCTCGGCACAATCT | CCACAGCTTTCTCCACTGCACT |

Supplementary Table S2. Baseline characteristics of NIAAA light drinkers with alcoholic-associated liver disease stratified by *ALDH2* rs671 genotypes

| **Patients** | **Overall (N = 38)** | ***ALDH2* rs671 genotypes** | | **W/χ2** | ***P*** |
| --- | --- | --- | --- | --- | --- |
|  |  | **Wild type (GG)**  **(n=16, 42.1%)** | **Variant (GA)**  **(n = 22, 57.9%)** |  |  |
| Age, Median (IQR) | 48 (42-56) | 50 (41-56) | 47 (42-57) | 181.5 | 0.882 |
| Male, n (%) | 38 (100.0) | 16 (100.0) | 22 (100.0) | 176 | 1.000 |
| BMI (kg/m²), Median (IQR) | 25.4 (23.2-27.3) | 24.9 (22.8-27.1) | 25.8(23.8-27.8) | 143.0 | 0.336 |
| CAP (db/m), Median (IQR) | 278.0 (258.0-293.0) | 268.2 (254.9-284.8) | 281.9 (261.5-298.8) | 124.0 | 0.129 |
| LSM (kpa), Median (IQR) | 6.0 (5.3-8.1) | 5.6 (5.2-6.2) | 7.4 (5.5-9.4) | 106.0 | **0.039** |
| Alcohol Consumption Patterns | |  |  |  |  |
| Type of beverage, n (%) |  |  |  | 0.3 | 0.422 |
| Beer | 10 (26.3%) | 3 (18.8%) | 7 (31.8%) |  |  |
| Spirits | 14 (36.8%) | 5 (31.2%) | 9 (40.9%) |  |  |
| Mixed (Beer and spirits) | 14 (36.8%) | 8 (50.0%) | 6 (27.3%) |  |  |
| Drinking frequency, n (%) |  |  |  | 113.0 | 0.054 |
| 1 time/month | 14 (36.8%) | 3 (18.8%) | 11 (50.0%) |  |  |
| 2-4 times/month | 8 (21.1%) | 5 (31.2%) | 3 (13.6%) |  |  |
| 2-3 times/week | 10 (26.3%) | 3 (18.8%) | 7 (31.8%) |  |  |
| ≥ 4 times/week | 6 (15.8%) | 5 (31.2%) | 1 (4.5%) |  |  |
| Alcohol intake (g/day), median (IQR) | 36.0 (28.5-55.0) | 33.0 (30.0-49.8) | 40.0 (24.3-55.0) | 175 | 0.988 |
| Standard drinks per week (drinks/week), median (IQR) | 4.9 (1.0-12.0) | 7.6 (2.4-12.0) | 2.4 (0.6-10.0) | 225.0 | 0.150 |
| Alcohol Intake Duration (years), median (IQR) | 17.5 (10.0-30.0) | 20.0 (13.8-30.0) | 12.5 (10.0-20.0) | 223.0 | 0.156 |
| Cumulative alcohol intake (kg), median (IQR) | 31.5 (8.8-74.0) | 62.2 (24.6-140.4) | 18.7 (7.6-55.9) | 243.0 | **0.049** |
| Laboratory Parameters, median (IQR) | |  |  |  |  |
| ALT (U/L) | 30.5 (19.8-41.2) | 35.6 (20.3-42.5) | 29.7 (14.6-37.8) | 206.5 | 0.375 |
| AST (U/L) | 26.8 (19.8-33.2) | 27.5 (20.0-33.3) | 26.7 (19.9-32.6) | 176.0 | 1.000 |
| GGT (U/L) | 63.0 (33.2-107.0) | 68.0 (42.5-103.3) | 55.8 (32.0-107.1) | 194.0 | 0.609 |
| ALP (U/L) | 80.8 (67.0-95.6) | 77.3 (63.9-89.6) | 82.1 (69.5-98.7) | 141.0 | 0.312 |
| Albumin (g/L) | 45.5 (43.9-47.5) | 46.5 (43.9-47.8) | 45.3 (44.0-47.0) | 213.0 | 0.280 |
| Total bilirubin (µmol/L) | 14.3 (11.0-19.6) | 15.4 (10.8-20.1) | 13.95 (11.7-18.5) | 184.0 | 0.824 |
| Total bile acid (µmol/L) | 2.5 (1.5-3.7) | 2.0 (1.2-3.0) | 3.0 (1.7-4.1) | 116.0 | 0.078 |
| Platelet count (10^9^/L) | 225.0 (200.0-255.0) | 217.0 (191.0-232.5) | 227.5 (203.0-270.5) | 136.0 | 0.243 |
| TG (mmol/L) | 2.1 (1.5-3.3) | 3.2 (1.6-3.9) | 1.8 (1.5-2.6) | 150.0 | 0.126 |
| LDL-C (mmol/L) | 76.1 (72.0-82.8) | 75.9 (71.9-81.2) | 76.8 (72.6-82.1) | 44.0 | 0.710 |
| Creatinine (µmol/L) | 3.6 (3.1-3.8) | 3.7 (3.3-4.1) | 3.3 (3.0-3.8) | 145.5 | 0.178 |
| APRI | 0.335 (0.254-0.402) | 0.354 (0.266-0.458) | 0.314 (0.242-0.375) | 192.5 | 0.636 |
| FIB-4 | 1.090 (0.870-1.290) | 1.087 (0.993-1.230) | 1.113 (0.766-1.477) | 169.0 | 0.848 |

Data are presented as median (IQR) or n (%), as appropriate. The ALDH2 rs671 genotypes were classified as the wild-type genotype (GG) and the heterozygous genotype (GA). W/χ² indicates the Wilcoxon rank-sum test statistic or chi-square test statistic, as appropriate. ALDH2, Aldehyde Dehydrogenase 2; ALP, Alkaline Phosphatase; ALT, Alanine Aminotransferase; APRI, Aspartate Aminotransferase to Platelet Ratio Index; AST, Aspartate Aminotransferase; BMI, Body Mass Index; CAP, Controlled Attenuation Parameter; FIB-4, Fibrosis-4 Index; GGT, Gamma-glutamyl Transferase; IQR, Interquartile Range; LDL-C, Low-density Lipoprotein Cholesterol; LSM, Liver Stiffness Measurement; NIAAA, National Institute on Alcohol Abuse and Alcoholism; TG, Triglyceride. Bold *P* values indicate statistical significance (*P* < 0.05).
